# Grado de satisfacción de estudiantes de odontología respecto al empleo de juegos didácticos en tiempos de pandemia por COVID-19

**DOI:** 10.21142/2523-2754-0903-2021-069

**Published:** 2021-10-06

**Authors:** Natalia Gutiérrez-Marín

**Affiliations:** 1 Departamento de Odontopediatría y Ortodoncia, Facultad de Odontología, Universidad de Costa Rica. San José, Costa Rica. natalia.gutierrez@ucr.ac.cr Universidad de Costa Rica Departamento de Odontopediatría y Ortodoncia Facultad de Odontología Universidad de Costa Rica San José Costa Rica natalia.gutierrez@ucr.ac.cr

**Keywords:** estudiantes, educación, odontología, difusión de innovaciones, students, education, dentistry, diffusion of innovation

## Abstract

**Objetivo:**

Determinar el grado de satisfacción de los alumnos respecto de la utilización de juegos didácticos en el curso de Odontología Infantil I, de la Facultad de Odontología, Universidad de Costa Rica.

**Métodos:**

Enfoque cualitativo, diseño descriptivo. Se crearon 5 juegos didácticos con la aplicación Educaplay, referentes a evaluación del riesgo cariogénico, patología general del niño, caries de la temprana infancia, remineralización dental y anatomía de piezas temporales; los juegos fueron implementados en el curso de forma alternada. Al finalizar el curso, se les envió vía correo institucional una encuesta a los 52 alumnos para valorar la utilización de los juegos.

**Resultados:**

38 estudiantes respondieron la encuesta en la cual el 97,4% consideró útil o muy útil la utilización de los juegos didácticos en las lecciones; además, el 65,8% indicó que los juegos habían ayudado mucho a mejora el aprendizaje y el 71,1% manifestó que las clases donde se aplicó el juego fueron más dinámicas. Además, al 92,1% de los encuestados les gustaría que se implementaran estas dinámicas en otros cursos.

**Conclusiones:**

Los estudiantes del curso de Odontología Infantil I mostraron un alto grado de satisfacción referente a la utilización de juegos didácticos durante las lecciones, los juegos los mantuvieron motivados.

## Abstract (en)

**Objetive:**

To determine the degree of student satisfaction regarding the use of didactic games in Infantile Odontology I at the School of Dentistry of the Costa Rica University.

**Methods:**

This was a descriptive study with a qualitative approach. Five didactic games were created using the Educaplay application and were implemented alternately: evaluation of cariogenic risk, general pathology of children, early childhood cries, dental remineralization and anatomy of temporal pieces. At the end of the course, a survey was sent by institutional mail to the 52 students to assess the use of the games.

**Results:**

38 students answered the survey, with 97.4% considering the didactic games useful or very useful in the lessons. Furthermore, 65.8% indicated that the games had greatly helped to improve learning and 71.1% stated that the classes in which the game was applied were more dynamic. In addition, 92.1% of the respondents would like this dynamic to be implemented in other courses.

**Conclusions:**

The students of Infantile Odontology I reported a high degree of satisfaction regarding the use of didactic games during the lessons, describing that the games maintain motivation.

## INTRODUCCIÓN

En los últimos años, las tecnologías de la información y la comunicación (TIC) han cobrado gran relevancia, ya que el desarrollo acelerado de *software* educativo ha transformado los componentes del proceso de enseñanza-aprendizaje y revolucionado los modelos pedagógicos tradicionales en nuevos espacios donde el tiempo y los recursos para el aprendizaje no están limitados. Las instituciones de educación superior han sufrido grandes transformaciones debido a que la virtualización ha generado soluciones tecnológicas dirigidas a adaptarse a esquemas más flexibles de enseñanza, en los que se han creado nuevos escenarios tecnológicos-educativos que requieren la implantación de la docencia virtual en el modelo educativo de la universidad tradicional ^1^.

Uno de los fines principales de la educación es generar procesos de aprendizaje por medio de los cuales los discentes puedan ejecutar diferentes actividades cognitivas que promuevan el desarrollo de estructuras mentales y la construcción del conocimiento ^2^. Entre estas actividades cognitivas, la utilización de los juegos didácticos en el aula apuesta por la innovación abierta que permite procesos activos en la construcción del conocimiento junto al aprendizaje por descubrimiento y significativo ^3^.

Las actividades que involucran el empleo de juegos diácticos son útiles, ya que el estudiante deja su posición pasiva y adopta una posición activa en el proceso de aprendizaje ^4^. Estos juegos se convierten en herramientas para promover el aprendizaje y transferir conocimiento por su capacidad de simular la realidad y ofrecer un escenario para cometer errores y aprender de ellos en la práctica. La educación lúdica establece una relación donde se permite el desarrollo permanente del pensamiento individual y un continuo intercambio con el pensamiento colectivo, a la vez que se le da relevancia al estudiante en su propio aprendizaje y se produce una retroalimentaicón inmediata ^5^^,^^6^. 

Las actividades lúdicas en el ámbito universitario están encaminadas a aumentar la motivación del alumnado a partir del uso de vivencias de juego donde se desarrollan habilidades, se disminuyen los esfuerzos cognitivos que se pudieran presentar y se acrecientan los diversos tipos de aprendizajes. Adicionalmente, los juegos didácticos favorecen la cooperación y competitividad entre los estudiantes, situaciones que benefician la obtención de los objetivos educativos planteados ^7^.

Algunas investigaciones respecto de la utilización de los juegos didácticos en la educación superior han dado buenos resultados. En un estudio con alumnos de la carrera de Ingeniería de Sistemas de la Universidad Técnica de Machala, emplearon la estructura del juego “quién quiere ser millonario” y obtuvieron un fortalecimiento del proceso de enseñanza-aprendizaje al emplear en el desarrollo de las lecciones una estrategia innovadora ^8^. Otro ejemplo se dio en la Escuela Preparatoria Regional de Atotonilco, de la Universidad de Guadalajara, donde en el curso de Química II se empleó la lúdica como estrategia didáctica para facilitar en el estudiante el entendimiento de la nomenclatura de la química orgánica, con resultados muy favorables ^9^. Por último, una investigación utilizó un juego denominado “Platform wars simulation” para mejorar la adquisición de competencias en el curso de contabilidad de gestión de la Facultad de Economía de la Universidad de Valencia. Los resultados obtenidos robustecen la evidencia de que los juegos didácticos pueden ser una vía para optimizar el aprendizaje ^3^. 

A nivel del área de la odontología, no se han encontrado reportes referentes a este tema, y con motivo de la suspensión de las lecciones presenciales debido a la pandemia producida por el virus SARS-CoV-2, los juegos didácticos podrían ser una opción innovadora para dinamizar los cursos. Es por lo anterior que el objetivo de este estudio fue determinar el grado de satisfacción de los alumnos respecto del uso de juegos didácticos en el curso de Odontología Infantil I de la Facultad de Odontología de la Universidad de Costa Rica.

## MATERIALES Y MÉTODOS

Se realizó una investigación cualitativa descriptiva en el 2020 para valorar el grado de satisfacción de los alumnos respecto de la utilización de juegos didácticos en el curso de Odontología Infantil I (O-2008), el cual se imparte en el VII ciclo de carrera de la licenciatura en Odontología de la Universidad de Costa Rica. El total de estudiantes matriculados fue de 52. Tradicionalmente, el curso era presencial con lecciones de 2 horas semanales, pero, debido a la suspensión de clases presenciales por la pandemia, el curso pasó a ser virtual con sesiones de 1 hora y 30 minutos durante 15 semanas. 

La investigación se desarrolló en 3 fases: la primera fue el diseño de los juegos didácticos que se emplearon de manera formativa, no sumativa. Los juegos se validaron con un grupo de estudiantes de odontología que no estaban llevando el curso. La segunda fase fue la aplicación de dichos juegos y la tercera fase estuvo formada por la creación y ejecución de una encuesta al estudiantado.

### Procedimiento de recogida y análisis de información

#### Fase 1. Elaboración de juegos didácticos

El curso de Odontología Infantil I consta de 10 temas de los cuales se seleccionaron los impares para realizar los juegos didácticos de manera que las actividades lúdicas fueran aplicadas de forma alternada. Los temas elegidos fueron los siguientes: evaluación del riesgo cariogénico, patología general del niño, caries de la temprana infancia, remineralización dental y anatomía de piezas temporales. 

Para la generación de los juegos didácticos, se eligió la aplicación Educaplay, la cual es gratuita y ofrece gran variedad de ejercicios que son accesibles desde cualquier dispositivo sin tener que instalar un programa. Además, los juegos quedan habilitados para ser ejecutados de forma ilimitada. 

**Figura 1:**
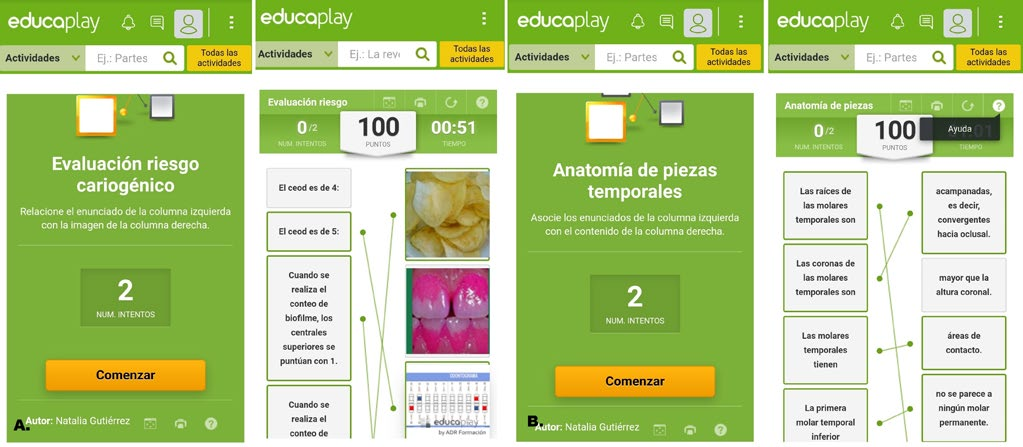
Juegos de asociación de columnas. A. Asociación de columnas e imágenes. B. Asociación de columnas y texto.

Se seleccionaron diferentes tipos de juegos para valorar cuál formato era el más aceptado. Se realizaron asociaciones de columnas usando imágenes y texto, lo cual consistía en relacionar un texto de la columna de la izquierda con el enunciado o la figura de la columna derecha. Si la persona asocia bien el enunciado, se genera una línea verde entre ellos, pero si la selección no fue la adecuada, se señalan en rojo y el sujeto tiene un segundo intento. Si se falla más de 2 veces, el juego se cierra, pero inmediatamente se puede volver a acceder y jugar de nuevo. Este formato se usó para los siguientes temas: evaluación del riesgo cariogénico (https://es.educaplay.com/recursos-educativos/5868600-evaluacion_riesgo_cariogenico.html); anatomía de piezas temporales (https://es.educaplay.com/recursos-educativos/5650369-anatomia_de_piezas_temporales.html) y caries de la temprana infancia (https://es.educaplay.com/recursos-educativos/6087645-caries_de_la_temprana_infancia.html) (Figura 1). 

Fuente: Elaboración propia

**Figura 2:**
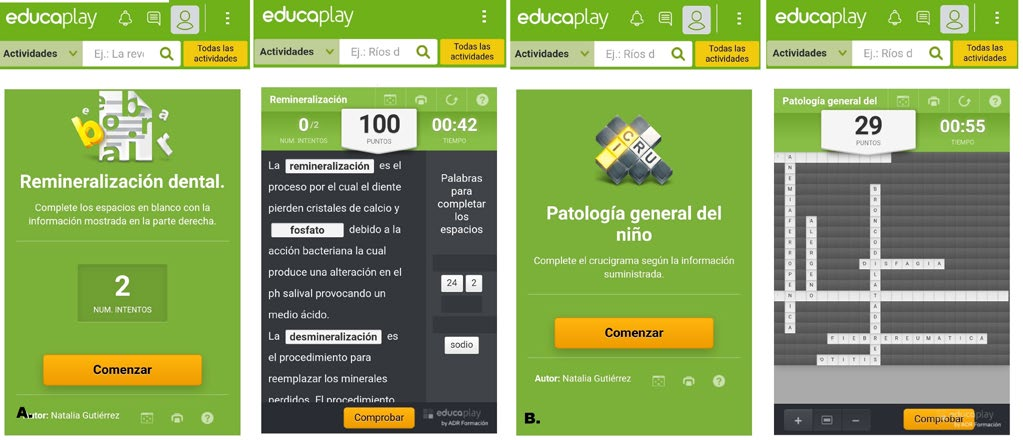
A. Juego de completar. B. Crucigrama.

Para el tema de remineralización dental, se empleó el formato de completar (https://es.educaplay.com/recursos-educativos/5924345-remineralizacion.html), en cual se presenta un texto con espacios en blanco y unas palabras que se deben colocar según el contexto. Finalmente, para el tópico de patología general del niño, se utilizó un crucigrama (https://es.educaplay.com/recursos-educativos/6179832-patologia_general_del_nino.html) en el que se les brinda como pista la letra inicial de la palabra que se solicita (Figura 2). 

Fuente: elaboración propia.

#### Fase 2. Aplicación de juegos didácticos

Las clases fueron impartidas de forma virtual durante 15 semanas. Cada 15 días, al finalizar la exposición del tema, se les brindaba a los estudiantes un enlace para que accedan al juego didáctico y lo resolvieran de forma individual. Luego de transcurridos unos 5 minutos, se compartía lo desarrollado en la sesión virtual y se aclaraban las dudas que podían presentarse. 

#### Fase 3: Ejecución de encuesta

Finalizado el curso, se creó una encuesta en SurveyMonkey (constituida por 8 preguntas cerradas y 1 abierta). La encuesta fue previamente validada y tenía un encabezado donde se explicaban su propósito y la confidencialidad, así como el tiempo que durarían en completarla y el asentimiento a participar. La encuesta fue enviada vía correo institucional para que el estudiantado valorara la implementación de los juegos didácticos y tuviera la oportunidad de expresar sus comentarios al respecto. Se dio una semana para que los discentes contestaran la encuesta; transcurrido ese tiempo, se les envió un recordatorio a los que no habían respondido y se habilitó por una semana más. 

Los datos se registraron en una hoja de cálculo de Excel (Microsoft, Inc., Redmond, WA, EE. UU.). Se ingresaron y se corrigieron las inconsistencias. Se realizó estadística descriptiva estableciendo la frecuencia absoluta y relativa de cada respuesta en la encuesta. Posteriormente, se utilizó la prueba de chi cuadrado para determinar si existía diferencia en la distribución de los estudiantes por sexo (p < 0,05, IC 95%). Todos los análisis se desarrollaron en el programa SPSS versión 22.0 (SPSS Inc., Chicago, IL, EE. UU.).

## RESULTADOS

De los 52 estudiantes matriculados en el curso, 38 contestaron la encuesta. El 97,4% consideró muy útil o útil el uso de juegos didácticos en algunas lecciones. Un 65,8% indicó que los juegos les ayudaron a mejorar el aprendizaje, mientras que un 71,1% consideró que los juegos dinamizaron las lecciones y solo un 7,9% señaló que no los motivaron. El 97,4% de los participantes prefirió las clases con juegos didácticos, y el tipo de juego preferido fue el de relacionar columnas utilizando texto e imágenes, mientras que la cantidad de preguntas en cada juego fue considerada como moderada por el 78,9% de los sujetos. Finalmente, a un 92,1% de los estudiantes les gustaría que se incluyan juegos didácticos en el curso de Odontología Infantil II. No hubo diferencia estadísticamente significativa en la distribución de los estudiantes por sexo en referencia a las respuestas dadas (Tabla 1).

**Tabla 1:** Preguntas y respuestas a la encuesta aplicada (N = 38)

	Total	Masculino	Femenino	p*
	n (%)	n (%)	n (%)	
¿Encontró útil la utilización de juegos didácticos en algunas lecciones del curso Odontología Infantil I?				0,949
Muy útil	15 (39,5%)	7 (43,7%)	8 (36,4%)	
Útil	22 (57,9%)	8 (50,0%)	14 (63,6%)	
Poco útil	1 (2,6%)	1 (6,3%)	0 (0%)	
¿Considera que los juegos didácticos ayudaron a mejorar el aprendizaje del curso Odontología Infantil I?				0,502
Mucho	25 (65,8%)	10 (62,5%)	15 (68,2%)	
Poco	12 (31,6%)	5 (31,2%)	7 (31,8%)	
Nada	1 (2,6%)	1 (6,3%)	0 (0,0%)	
¿Piensa que el empleo de juegos didácticos hizo que las clases de Odontología Infantil I fueran más dinámicas?				0,065
Mucho	27 (71,1%)	9 (56,4%)	18 (81,8%)	
Poco	10 (26,3%)	6 (37,5%)	4 (18,2%)	
Nada	1 (2,6%)	1 (6,3%)	0 (0,0%)	
¿Considera que el uso de juegos didácticos aumentó su motivación en el curso de Odontología Infantil?				0,871
Mucho	18 (47,4%)	8 (50,0%)	10 (45,5%)	
Poco	17 (44,7%)	6 (37,5%)	11 (50,0%)	
Nada	3 (7,9%)	2 (12,5%)	1 (4,5%)	
Le gustaron más las clases:				0,421
Con juego didáctico	37 (97,4%)	15 (93,7%)	22 (100%)	
Sin juego didáctico	1 (2,6%)	1 (6,3%)	0 (0,0%)	
Considera que la cantidad de preguntas en cada juego didáctico fue:				0,770
Alta	0 (0,0%)	0 (0,0%)	0 (0,0%)	
Moderada	30 (78,9%)	13 (81,3%)	17 (77,3%)	
Baja	8 (21,1%)	3 (18,7%)	5 (22,7%)	
¿Cuál tipo de juego didáctico le gustó más?				0,818
Relacionar columnas usando texto	11 (28,9%)	5 (31,3%)	6 (27,3%)	
Complete	0 (0,0%)	0 (0,0%)	0 (0,0%)	
Crucigrama	10 (26,4%)	4 (25%)	6 (27,3%)	
Relacionar columnas usando texto e imágenes	17 (44,7%)	7 (43,7%)	10 (45,4%)	
¿Le gustaría que en el curso de Odontología Infantil II se incluyeran juegos didácticos?				0,562
Sí	35 (92,1%)	14 (87,5%)	21 (95,5%)	
No	3 (7,9%)	2 (12,5%)	1 (4,5%)	

Prueba de chi cuadrado

Algunos de los comentarios de los estudiantes en la pregunta abierta fueron los siguientes: “es una muy buena herramienta para fomentar la atención en clase”, “es una forma de aprender que no es mediante un examen donde uno está más relajado y ordena mejor las ideas que bajo presión”, “los juegos ayudan a repasar lo visto en clase y en ningún otro curso se usó algo así”, “excelente iniciativa, aunque a veces no hay tiempo de completar los juegos didácticos en clase, pero se pueden realizar en otro momento”, “parte de que los juegos resulten atractivos al estudiante para repasar es que ni tienen valor, y se hacen con menos estrés, por lo que se termina aprendiendo más”, “la verdad que esta iniciativa de los juegos ayudó a la hora de repasar ya que se podían volver a hacer”, “forma distinta de mantener el interés de los estudiantes y no solo llamarlos para contestar preguntas por medio del micrófono”. Solo un estudiante comentó que “los juegos habían sido poco útiles”.

## DISCUSIÓN

Al ser la educación un proceso dinámico, se requiere que los docentes realicen innovaciones referentes a la forma en la que imparten sus lecciones. La implementación de juegos didácticos en estudiantes de odontología fue muy bien aceptada por los discentes, lo cual aporta evidencia para generar nuevas actividades que dinamicen las lecciones .

Los juegos didácticos constituyen herramientas útiles, ya que tienen la capacidad de transformar aprendizajes no comprendidos y convertirlos en oportunidades de enseñanza importantes y llamativas ^10^. Respecto de la utilidad de la implementación de juegos didácticos en algunas lecciones del curso Odontología Infantil I, el 39,5% de los encuestados la encontró muy útil, mientras que el 57,9% la consideró útil; más aún, el 97,4% indicó que le gustaron más las clases en las que se emplearon los juegos didácticos. Un resultado similar se dio en una investigación realizada en Lima, Perú, donde se utilizó Educaplay para desarrollar las capacidades de comprensión y producción de textos en inglés, y el 52,5% de los alumnos indicaron que los juegos habían sido útiles para reforzar dichas capacidades ^11^.

La motivación de los discentes es un aspecto muy importante en los procesos educativos, pues se considera que, entre más motivado esté el alumno, más aprenderá y su aprendicaje será significativo ^12^. Motivar y dinamizar las lecciones son algunos de los objetivos que se establecen a la hora de implementar juegos didácticos. En esta investigación, el 71,1% de los encuestados indicó que la utilización de los juegos dinamizó mucho el curso, mientras que solo el 7,9% de los sujetos señaló que la utilización de los juegos había aumentado poco la motivación. Lo anterior concuerda con un estudio realizado con estudiantes de pregrado de la Universidad de La Sabana, donde se utilizó la plataforma de Educaplay para realizar diversos juegos didácticos, los cuales aumentaron la motivación en los estudiantes y mejoraron la dinámica de la lección ^13^. Por su parte, algunos investigadores también hacen referencia a que los juegos didácticos aumentan la motivación en el estudiantado, quienes logran incrementar su vínculo con el aprendizaje al sentirse complacidos con las dinámicas realizadas ^14^. 

Cuando se consultó a los estudiantes si consideraban que los juegos didácticos ayudaron a mejorar el aprendizaje del curso Odontología Infantil I, el 65,8% respondió que mucho, lo cual es un aspecto importante porque esto podría influir de forma positiva en el rendimiento académico de los alumnos. Una revisión sistemática sobre las implicaciones de la gamificación en educación superior indica que los alumnos que se ven involucrados en entornos de aprendizaje gamificados mejoran su aprendizaje ^15^. Más aún, una investigación evidenció que el uso de Educaplay mejoró de forma estadísticamente significativa las capacidades en el área de inglés en los estudiantes de la Institución Educativa San Antonio de Jicamarca ^16^. En España se realizó otro estudio que arrojó resultados positivos, en el cual se empleó Educaplay para fortalecer la comprensión lectora de textos en español. El autor, además, concluyó que el uso de la plataforma favoreció el proceso de enseñanza-aprendizaje debido, probablemente, a que las nuevas generaciones conciben las herramientas tecnológicas como parte fundamental de sus vidas ^17^.

Respecto del tipo de juegos empleados, la relación de columnas usando texto e imágenes fue la preferida (44,7%), seguida por los crucigramas (26.4%). Adicionalmente, la mayoría de los estudiantes (78,9%) indicaron que la cantidad de preguntas en cada juego fue moderada. Educaplay permite incorporar imágenes, lo cual resulta muy útil en el área de la odontología, porque se pueden utilizar radiografías o fotografías que muestren distintas patologías o casos clínicos. Por su lado, los crucigramas sirven como instrumentos para la estimulación del pensamiento crítico, la resolución de problemas y el desarrollo de habilidades relacionadas con la adquisición de terminología ^18^. En un estudio, se utilizó con éxito la aplicación Educaplay para contribuir al aprendizaje de las biomoléculas en los estudiantes de bachillerato de la Unidad Educativa Andrés F. Córdova; ellos emplearon los crucigramas como parte de las herramientas para innovar en la forma de impartir las lecciones ^19^. 

En cuanto al uso de los juegos didácticos en el curso de Odontolgía Infantil II, el 92,1% de los estudiantes lo recomendó, lo cual se relaciona con los comentarios externados por los discentes, la mayoría de los cuales coincidió en que los juegos didácticos les permitieron repasar los contenidos vistos en clase, a la vez que los ayudó a mantener el interés en las lecciones. 

En ninguna de las preguntas hubo una diferencia estadísticamente significativa entre hombres y mujeres, lo cual es un aspecto importante, ya que sugiere que los juegos didácticos pueden emplearse en diversos grupos de estudiantes.

Una de las limitaciones del estudio fue que los juegos se aplicaron a solo un curso, por lo que en el futuro se planea la incorporación de juegos didácticos generados con diferentes aplicaciones en otros cursos teóricos de la carrera de Odontología en la Universidad de Costa Rica.

## CONCLUSIÓN

Los juegos didácticos pueden ser herramientas muy útiles para generar aprendizajes significativos, ya que los alumnos aprenden practicando. Los estudiantes del curso de Odontología Infantil I mostraron un alto grado de satisfacción respecto de la utilización de juegos didácticos durante las lecciones, lo cual representa un hecho importante para tomar en cuenta a la hora de diseñar las lecciones y crear innovaciones que ayuden a construir los procesos de enseñanza-aprendizaje, máxime en estos tiempos de pandemia en los que la motivación de los estudiantes es vital para mantenerlos inmersos en la adquisición de los conocimientos y competencias necesarias para desenvolverse en el mundo profesional. 

## Fuente de financiamiento:

Autofinanciado
